# Preserving *Porphyra* *umbilicalis* and *Saccharina latissima* as Silages for Ruminant Feeding

**DOI:** 10.3390/ani10111957

**Published:** 2020-10-23

**Authors:** Margarita Novoa-Garrido, Carlos Navarro Marcos, María Dolores Carro Travieso, Eduarda Molina Alcaide, Mogens Larsen, Martin Riis Weisbjerg

**Affiliations:** 1 Faculty of Biosciences and Aquaculture, Nord University, P.O. 1490, 8049 Bodø, Norway; 2 Departamento de Producción Agraria, Escuela Técnica Superior de Ingeniería Agronómica, Alimentaria y de Biosistemas, Universidad Politécnica de Madrid, Ciudad Universitaria, 28040 Madrid, Spain; navarro-88@hotmail.com (C.N.M.); mariadolores.carro@upm.es (M.D.C.T.); 3 Estación Experimental del Zaidin (Consejo Superior de Investigaciones Cientificas), Profesor Albareda, 1, 18008 Granada, Spain; molina@eez.csic.es; 4 Department of Animal Science, Aarhus University, AU Foulum, Blichers Allé 20, 8830 Tjele, Denmark; mogens.larsen@anis.au.dk (M.L.); martin.weisbjerg@anis.au.dk (M.R.W.)

**Keywords:** seaweed, ensiling, chemical composition, pH, in vitro rumen fermentation

## Abstract

**Simple Summary:**

Seaweeds are underutilized resources worldwide that could be used in both the food and the feed markets. However, seaweeds decompose quickly mainly due to their high water content and therefore cost and energy effective preservation methods must be explored. Silage is a low-energy input method to preserve forage crops widely used worldwide, but the ensilability of seaweeds has been little investigated. In this study, we assessed different procedures to ensile *Porphyra umbilicalis* and *Saccharina latissima,* including the washing and pre-wilting of the seaweeds before ensiling and the addition of formic acid. The chemical composition and in vitro ruminal fermentation of the obtained silages were determined to investigate their potential use as ruminant feed. Seaweeds did not undergo the typical silage fermentation, characterized by high production of lactic acid, as usually observed for terrestrial crops, and silage characteristics were variable depending on the seaweed species. All silages with formic acid as silage additive had pH values below the recommended value of 4.0, whereas those without formic acid had pH values greater than 4.50. The addition of formic acid also increased the ruminal degradability of the silages. More studies are needed to identify optimal ensiling conditions for seaweeds.

**Abstract:**

The study analyzed the characteristics, chemical composition, and in vitro gas production kinetics of *Porphyra umbilicalis* and *Saccharina latissima* silages. Each seaweed was ensiled in vacuum bags (three bags/silage) following a 2 × 3 factorial design, with two pre-treatments (unwilted or pre-wilted) and three silage types: unwashed seaweed ensiled without additive; seaweed washed and ensiled without additive; and seaweed washed and ensiled with 4 g of formic acid (FAC) per kg seaweed. Silages were kept for 3 months in darkness at 20 °C. Pre-wilting prevented (*p* < 0.001) effluent formation and reduced (*p* ≤ 0.038) the production of NH_3_-N and volatile fatty acids for both seaweeds. Both pre-wilting and washing increased (*p* < 0.05) the ruminal degradability of *P. umbilicalis* silages but not of *S. latissima* silages. The pH of the FAC-treated silages was below 4.0, but ranged from 4.54 to 6.23 in non FAC-treated silages. DL-lactate concentrations were low (≤23.0 g/kg dry matter) and acetate was the predominant fermentation product, indicating a non-lactic fermentation. The estimated ruminal degradability of the *P. umbilicalis* and *S. latissima* silages was as average, 59.9 and 86.1% of that for high-quality rye-grass silages, respectively, indicating a medium-low nutritional value of these seaweed silages for ruminants.

## 1. Introduction

Seaweeds are still underutilized resources worldwide for both human food and animal feed markets, and there is therefore an increasing interest in expanding the array of applications of seaweeds to surpass the USD 7 billion annual value of today’s seaweed industry [1,2]. Seaweeds’ growth is primarily light rather than temperature dependent, which explains their clearly seasonal growth [3,4]. This is of particular importance in most northern regions of the temperate climate zone and in the frigid climate zone. Decomposition processes start quickly after harvest due to high water content in seaweed [5], and it is therefore necessary to develop technologies that allow storage throughout the year for seaweeds to become a resource of interest for animal feed industry. A feasible storage method should be independent of harvest time, cost and energy efficient, and should preserve the content in nutrients and bioactive compounds of the seaweed biomass. Several technologies can be considered for seaweed preservation. Drying or freezing are two available technologies, but both are high-energy demanding methods. Silage is a low energy input method to preserve forage crops for livestock feeding widely used worldwide. The principle of silage is the conversion of water-soluble carbohydrates present in the crops to organic acids, mainly lactic acid, by epiphytic lactic acid bacteria under anaerobic conditions, resulting in a pH drop that inhibits the growth of undesirable microorganisms and reduces feed deterioration. When the epiphytic lactic bacteria population in the crop is not sufficient to successfully reduce the pH, the silage process can be enhanced by the use of inoculants (i.e., lactic acid bacteria) and/or additives, such as organic acids [6].

Seaweeds differ from terrestrial crops in some key characteristics, which may challenge the silage process. Seaweeds present sugar alcohols (i.e., mannitol) and can have high content in non-fermentable sugars such as 3,6-anhydrogalactose in red seaweeds, and alginic acid in brown seaweeds [7]. Furthermore, seaweeds usually have high pH, high content of water and ash, and present low or negligible epiphytic lactic acid bacteria population [8,9], and all these factors can contribute to inhibiting the lactic acid fermentation [10]. The presence of bioactive compounds [11,12], together with typical seaweed polysaccharides such as sulphated fucans and sodium alginate, might also be limiting factors for the silage fermentation processes [13,14,15]. Moreover, seaweeds can have high levels of non-digestible dietary fiber [16,17] that can be fermented in the rumen [18,19], and therefore can be more efficiently used in ruminant than in non-ruminant feeding.

Seaweeds’ composition varies widely depending on types and species [18,20,21], and these variations may affect the ability to ensile. Although the nutritive value of different seaweeds species has been investigated [19,22,23], research on how to ensile seaweeds for animal feeding is still scarce [24,25,26], and it is necessary to adapt and further develop the existing knowledge to each of the indigenous seaweed species. Understanding the silage of seaweeds as a cost-effective preservation method is crucial in order to continue the development of the seaweeds industry. Our hypothesis is that silage can be a feasible preservation method for seaweeds, and that silage procedure may change the chemical composition and nutrients availability of the seaweed silages. Thus, the aim of this study was to assess the feasibility of silage preservation of two commercially interesting seaweed species (*Porphyra umbilicalis* and *Saccharina latissima*), either unwilted or pre-wilted, and with or without addition of formic acid (FAC). The in vitro rumen fermentation of seaweed silages was also evaluated using the gas production technique. *Porphyra umbilicalis* was selected for the study because it has high N content and it can supply a high amount of digestible protein to the small intestine of ruminants [20], but information on its ensilability is not yet available. The ensilability potential of *S. latissima*, a low-fiber content seaweed, has been tested in previous studies [24,25], but the present study assess new silage treatments that might improve the silage process. In addition, *S. latissima* is successfully cultivated in sustainable aquaculture systems in Norway and other countries along the North Atlantic Ocean [27].

## 2. Materials and Methods 

### 2.1. Seaweed Collection and Silage Preparation

Biomass of *P. umbilicalis* and *S. latissima* was collected from wild stocks by hand picking at low tide in the Bodø municipality (Norway), in June 2016 (67°16′57″ N 14°22′30″ E). Both seaweeds were collected in mid-June at expected stage of high quality (high nitrogen (N) content and low neutral detergent fiber (NDF) content) [19]. The seaweed biomass was maintained in tanks with running seawater until further processing within two days after collection. The excess water was drained manually, and the biomass was cut into pieces of about 2 cm × 3 cm using scissors before ensiling. 

For each seaweed, silage treatments followed a 2 × 3 factorial design, with two pre-wilting treatments (either unwilted or pre-wilted) and three silage types: seaweed unwashed and ensiled without any additive (control; CON), seaweed washed and ensiled without any additive (WAS) and seaweed washed and ensiled with 4 g of FAC per kg seaweed (WASFAC). For the pre-wilting treatment, 1 kg of each seaweed was weighed in netting bags which were placed into an air-forced oven at 37 °C, and weighed every 2 h until reaching a previously calculated weight based on a dry matter (DM) content of 300 g/kg. The targeted weight was calculated from earlier DM determinations of both harvested seaweeds in our laboratory [19]. The washing process of seaweeds performed for WAS and WASFAC silages was done to reduce surface salts and it consisted of three sequential brief baths (10 s) in plastic containers with water of decreasing salinity: seawater, a seawater:freshwater mixture (30:70), and finally freshwater.

The seaweed was chopped up, packed (1 kg) in vacuum bags (Lavezzini, Fiorenzuola d’Arda, Italy; dimensions 20 × 60 cm), and sealed with the aid of an automatic vacuum-packing machine (model Elix; Lavezzini, vacuum pump 20–24 l/min). The machine was equipped with a heat-sealing mechanism, and the bags were automatically sealed after air extraction. For the pre-wilted silages, the whole content of each netting bag was introduced in one vacuum bag. Three bags were prepared for each treatment, making a total of 18 silage bags for each seaweed (2 pre-wilting treatments × 3 silage types × 3 replicates). Silages were stored in darkness for 3 months at room temperature (20 °C), and afterwards the silage process was stopped by freezing. After thawing, 100 g of silage was diluted with 750 g demineralized water and homogenized in a Waring blender (Waring 24CB10; Waring Commercial, New Hartford, CT, USA) for 80 s (2 times × 40 s with a break of 1 min in between). The homogenate was then centrifuged for 10 min at 2500× *g*, and the pH of the supernatant (silage extract) was immediately measured (Meterlab PHM 220, Radiometer, Brønshøj, Denmark). Thereafter, subsamples of silage extracts were stored frozen (−20 °C) either without stabilization or stabilized with meta-phosphoric acid (5% final concentration in the sample). The rest of the silage was frozen, freeze-dried, and ground through a 1 mm sieve (ZM 200 mill, Retsch GmbH, Haan, Germany) before analyses of chemical composition and in vitro incubations. For processing of silages with effluent (free juice), juice and solids were separated using a kitchen sieve, and both fractions were weighed for calculations of effluent fraction and for recomposing representative samples for extraction and DM determination based on weight proportions. Recomposed samples were used for both chemical analyses and in vitro incubations.

### 2.2. Ruminal Fluid Donors and In Vitro Incubations

Four adult rumen-fistulated Lacaune sheep (64.2 ± 1.95 kg body weight (BW)) were used as donors of ruminal fluid for the in vitro incubations. Sheep were managed in accordance with the Spanish guidelines for experimental animal protection. Experimental procedures for rumen content sampling were approved by the Institutional Animal Care and Use Committee of the Comunidad Autónoma de Madrid of Spain (Approval number PROEX 035/17). Animals were individually housed, had free access to fresh water, and were fed twice daily (8:00 and 18:00) a 2:1 grass hay:concentrate diet at a rate of 45 g DM/kg BW^0.75^. The diet contained 114, 365 and 160 g of crude protein (CP), NDF and acid detergent fiber (ADF) per kg DM, respectively. 

Samples of each seaweed (unwashed and washed) and of lyophilized seaweed silages were used as substrates for in vitro incubations using batch cultures. In addition, two samples of perennial ryegrass silages (early and late cut) were incubated as reference feeds for comparison of gas production values. Rumen contents were collected from each sheep before the morning feeding, strained through four layers of cheesecloth into previously warmed (39 °C) thermal flasks, and immediately transported to the laboratory. The fluid of each sheep was independently mixed with a pre-warmed (39 °C) culture medium ([28]; without trypticase) in a proportion 1:4 under CO_2_ flushing, resulting in the incubation of each sample with four different inocula (ruminal fluid from different sheep). Samples (200 mg of DM) of each tested feed were weighed into 60-mL glass vials. Vials were filled with 20 mL of the rumen fluid–culture medium mixture using a peristaltic pump (Watson-Marlow 520UIP31; Watson-Marlow Fluid Technology Group, Cornwall, UK), sealed with rubber stoppers, and incubated at 39 °C for 120 h. Gas production was measured at 3, 6, 9, 12, 15, 22, 26, 31, 36, 48, 58, 72, 96 and 120 h using a plastic syringe and a pressure transducer (Delta Ohm DTP704-2BGI, Herter Instruments SL, Barcelona, Spain). In order to prevent gas accumulation, the gas produced at each sampling time was released until the pressure inside the vial reached zero. Two blanks (vials without sample) were used for each inoculum to correct gas production values for endogenous production. 

The potential in vitro DM degradability (DMPD) of each tested feed was determined in order to estimate the DM effective degradability (DMED). Samples (300 mg) of each feed were weighed in triplicate into polyester bags of 30 µm of pore size (Ankom Corp #57, Ankom Technology Corp., Fairport, NY, USA). Bags were incubated in a 1:4 mixture of ruminal fluid and the incubation medium of Goering and Van Soest [28] using an Ankom Daisy II incubator (Ankom Technology Corp.) at 39 °C under continuous rotation for 120 h. A mixture of equal parts of the ruminal fluid from each sheep was used as inoculum. After 120 h, bags were washed with cold water, dried at 60 °C for 48 h, and weighed to calculate PDMD.

### 2.3. Chemical Analyses

Silage extracts stabilized with meta-phosphoric acid were analyzed for volatile fatty acids (VFA), DL-lactate, NH_3_-N, alcohols, nonpolar amino acids, benzoate, and γ-amino-butyrate concentrations as described by Kristensen et al. [29]. Glucose and L-lactate concentrations in the extracts were analyzed using membrane-immobilized substrate specific oxidases (D-glucose and L-lactate oxidase, respectively; YSI 2900; YSI Inc., Yellow Springs, OH, USA). The potential inhibition of membrane L-lactate oxidase by seaweed compounds was tested by spiking 0, 3.75, 7.5, and 15 mM L-lactate in four replicas to extract samples of two *Saccharina* and two *Alaria* silages with zero to 2.5 mM measured L-lactate. The recovery of added L-lactate was 106.5 ± 0.48% indicating no inhibition of membrane oxidase by seaweed compounds.

All tested feeds were analyzed for DM, ash, N, NDF, ADF, lignin, and acid detergent insoluble N (ADIN) following the Association of Official Analytical Chemists [30] methods as detailed by Marcos et al. [31], with the exception that DM content in silages was determined by freeze-drying. Sodium sulphite was used in the sequential analysis of NDF, ADF and lignin, and ash-free values are reported.

### 2.4. Calculations and Statistics

Data of gas production were fitted to the exponential model Gas = PGP (1 − e ^− (c (t − *lag*))^) using the Proc NLIN of the Statistical Analysis System (SAS; [32]) by an iterative least squares procedure. In this model, PGP is the potential gas production (asymptotic value), *c* is the fractional rate of gas production, *lag* is the time before starting gas production, and t is the time of gas measurement. The average gas production rate (AGPR) was calculated as AGPR = PGP *c/*[2 (ln2 + *c lag*)] and it was defined as the rate between the incubation start and the time at which half PGP is reached [21,31]. The DMED was estimated as DMED = [(DMPD × *c*)/(*c* + *K*p)] e ^(−^*^kp^* ^× lag)^ assuming a rumen particulate outflow (Kp) of 3% per h, which is characteristic for sheep fed at maintenance level [31,33].

When data from both seaweeds were analyzed together, seaweed × pre-wilting treatment × silage type interactions (*p* < 0.05) were observed for most variables. Therefore, data were analyzed separately for each seaweed as a 2 × 3 factorial design using the Proc GLM of SAS [32]. The model included two pre-treatments (pre-wilted or not), three silage treatments (control, WAS and WASFAC) and the interaction pre-wilting × silage type as main effects. Parameters of gas production were analyzed using the Proc MIXED of SAS [32] as a mixed model in which the effects of pre-wilting, silage type, and their interaction were considered fixed and that of inoculum (ruminal fluid from four sheep) was considered random. When a significant effect (*p* ≤ 0.05) of silage type was detected, differences among means within each pre-wilting treatment were tested using the Tukey’s multiple comparison test.

## 3. Results and Discussion

### 3.1. Chemical Composition and In Vitro Gas Production Parameters of the Seaweeds Pre-Ensiling

The chemical composition and gas production parameters of fresh *P. umbilicalis and S. latissima* are presented in Table 1. Both seaweeds had low DM and high ash content, which agrees with previous studies [9,10,19] on the same seaweed species harvested in the same area. As expected, rinsing the seaweeds with fresh water reduced their ashes content and this should therefore be a first step in the post-harvest silage procedure, since high ash content negatively affects digestibility and silage process [15]. Although the two seaweeds differed in their N content, the proportion of ADIN was lower than 4 g/100 g of total N in both of them. The ADIN was considered an indicator of N availability by some ruminant feeding systems, although the validity of the analytical method has been recently questioned [34]. The low ADIN proportion in *P. umbilicalis* is consistent with the low indigestible proportion of CP reported previously for this seaweed [20], indicating that it can supply a high amount of digestible protein to the small intestine in ruminants [20]. Both seaweeds differed in their NDF content, which was greater for *P. umbilicalis* than for *S. latissima*, whereas the opposite was observed for ADF content. In agreement with previous reports [35,36], lignin was not detected in any of the two fresh seaweeds, although low lignin concentrations in some seaweeds have been reported [25].

The lower NDF proportion of *S. latissima* compared with *P. umbilicalis* agrees well with the greater PGP, *c*, AGPR and DMED values observed for *S. latissima* (2.3, 2.0, 4.1 and 1.5 times greater than in *P. umbilicalis*, respectively; values averaged for unwashed and washed seaweeds). Gas production is directly related to the amount of organic matter fermented by rumen microorganisms [37] and NDF is less extensive and rapidly fermentable than other chemical fractions such as sugars and non-structural carbohydrates. However, previous studies have reported lower digestibility for brown seaweeds in comparison with red seaweeds [9,38], possibly related to the presence of poorly digestible sulphated polysaccharide- and polyphenols-protein complexes typical in brown seaweeds [39,40]. The high DMPD values observed for both seaweeds are in accordance with previous studies [19,21].

### 3.2. Characteristics of the Seaweed Silages

The characteristics of the *P. umbilicalis* and *S. latissima* silages are presented in Table 2 and Table 3, respectively. As expected, the DM content was greater (*p* < 0.001) in the pre-wilted than in the unwilted silages for both seaweeds, but pre-wilting × silage type interactions were detected (*p* < 0.001 and 0.020 for *P. umbilicalis* and *S. latissima*, respectively). No differences (*p* > 0.05) in DM content were detected among the unwilted silages of both seaweeds, but when seaweeds were pre-wilted before ensiling the CON and WAS silages had the greatest (*p* < 0.05) DM content for *P. umbilicalis* and *S. latissima*, respectively. The DM content of all unwilted silages was below 190 g/kg, and was similar to that of the fresh seaweeds (Table 1). For the pre-wilted silages, the seaweeds were dried to achieve a target content of 300 g DM/kg, but the actual content was greater than that in all samples. A minimum DM content of 250 g/kg has been proposed as necessary for ensuring a good silage process [41], but the DM of the fresh seaweeds was lower than 216 g/kg (Table 1) and that could have affected the silage process negatively in the unwilted silages. 

Apart from preserving the nutritional value of the ensiled feed to the greatest extent possible, the handling of silages must be convenient for the silage of seaweeds to be of interest for the industry, and the presence of effluent complicates silage management. Effluent release usually occurs when the initial forage DM content is below 250–300 g/kg [41], causing a loss of nutrients and contribution to environmental pollution. Silage effluent is formed from the surface moisture of the plants and cell juice, which is released due to cell lysis during silage and washes out valuable highly-digestible compounds such as soluble carbohydrates, minerals, organic acids and alcohols [42]. Because of the low DM content of the seaweeds, effluents were present in all unwilted silages of both seaweeds, being greater for *P. umbilicalis* than for *S. latissima* (25.5 and 10.8% of total weight, respectively; values averaged across silage types). In contrast, no effluent was observed in any of the pre-wilted silages, which is consistent with the greater (*p* = 0.001) DM content of these silages compared with those unwilted.

For both seaweeds, the pH values in the pre-wilted silages were greater (*p* < 0.001) than in those unwilted, and pre-wilting × silage type interactions were detected (*p* < 0.001). As expected, the addition of FAC decreased the pH in all silages (*p* < 0.05) resulting in pH values below 4.0. According to Woolford and Pahlow [43], a pH lower than 4.2 is required for an effective silage process of samples containing about 200 g DM/kg, but due to the low DM content of the seaweeds even lower pH values might be required to prevent clostridial fermentation and the production of butyric acid. In the present study, only the FAC-silages reached the required pH. The pH of the pre-wilted silages without FAC (CON and WAS) was greater than the values reported by Cabrita et al. [25] for *S. latissima* silages when the seaweed was pre-wilted at 18–20 °C for 24 h (4.48 and 4.10 for silages with and without a microbial inoculant, respectively). Our values were also greater than those observed in *S. latissima* silages by Campbell et al. [26] when the seaweed was pre-wilted for 24 h (3.90) and by Herrman et al. [44] when the seaweed was not pre-wilted before ensiling (3.80). To our best knowledge this is the first report on *P. umbilicalis* silages, but the pH values (5.75 and 4.92 for pre-wilted CON and WAS, respectively) were in the range of those reported in previous studies [15,16] for silages of pre-wilted *Ulva rigida* (5.10), *Gracilaria vermiculophylla* (5.20) and *Fucus vesiculosus* (4.90) without any silage additive. Differences observed among seaweeds may be due to different content in easily fermentable components, but also to the silage conditions used in the different studies. As already discussed, ensiling seaweeds is challenging due to their low levels of water-soluble carbohydrates and of epiphytic lactic acid bacteria [44] and high buffering capacity [45], that can cause the poor reduction in pH during the silage fermentation process [44,46].

Concentrations of NH_3_-N were greater (*p* < 0.001) in the unwilted than in pre-wilted silages for both seaweeds, and CON silages showed the greatest (*p* < 0.05) NH_3_-N concentrations except for the pre-wilted silages of *P. umbilicalis* which showed no differences among them. The concentration of NH_3_-N is a common indicator of the proteolytic activity during silage fermentation [41], and our results indicate that pre-wilting decreased the proteolysis in both seaweeds. In general, the NH_3_-N concentrations were low, especially in the pre-wilted silages (<0.80 g/kg DM), suggesting a low proteolytic activity. When the NH_3_-N content was expressed as proportion of total N content in the silages, the NH_3_-N in unwilted *S. latissima* silages was 6.61, 3.24 and 3.39% of total N content for CON, WAS and WASFAC, respectively, and only 2.42, 0.89 and 1.88% of total N for the CON, WAS and WASFAC pre-wilted silages. These proportions are lower than the values ranging between 4.7 and 6.7% of total N reported previously for pre-wilted *S. latissima* silages [25,26]. The NH_3_-N content in the unwilted *P. umbilicalis* silages, expressed as % of total N, reached greater values (15.1, 7.32 and 5.11% for CON, WAS and WASFAC, respectively), but most of them were lower than the 10.7 to 100% values reported in other studies for pre-wilted silages of *Ulva rigida*, *F. vesiculosus* and *G. vermiculophylla* [25,26]. Content of NH_3_-N in *P. umbilicalis* pre-wilted silages was low, reaching only 0.49, 2.19 and 1.17% of total N for CON, WAS and WASFAC, respectively. In accordance with the greater NH_3_-N concentrations observed for the unwilted silages, free amino acids content was in general greater in the unwilted silages compared with those pre-wilted for both seaweeds. Significant pre-wilting × silage type interactions were detected for valine in *P. umbilicalis* and for most amino acids in *S. latissima* silages. For *P. umbilicalis*, the WAS silage had greater (*p* < 0.05) concentrations of free amino acids than CON and WASFAC, whereas for *S. latissima* CON silages had the greatest content in free amino acids. 

Glucose concentrations were low in the *P. umbilicalis* silages (<0.760 g/kg DM) and were not affected (*p* ≥ 0.110) by either pre-wilting or silage type, but glucose concentrations in *S. latissima* silages were greater (6.22 to 78.8 g/kg DM) and were affected (*p* ≤ 0.002) by both pre-wilting treatment and silage type. *S. latissima*, also known as “sugar kelp”, is rich in high fermentable sugars [44], which might help to explain the greater glucose concentrations in its silages. Pre-wilting decreased (*p* = 0.002) glucose concentrations in *S. latissima* silages. Similarly, the use of FAC reduced (*p* < 0.05) the glucose concentration in the silages compared with those untreated with FAC.

Pre-wilting tended to decrease (*p* = 0.070) DL-lactate concentrations in *P. umbilicalis* silages, and the WAS silages showed greater (*p* < 0.05) concentrations of DL-lactate than CON and WASFAC regardless of pre-wilting. Pre-wilting also decreased (*p* < 0.001) DL-lactate concentrations in *S. latissima* silages, but a pre-wilting × silage type interaction was detected (*p* = 0.005). For the unwilted silages, WASFAC had lower (*p* < 0.05) DL-lactate concentrations than CON and WAS, whereas no differences among silage types were detected in the pre-wilted silages. In agreement with our results, low lactate concentrations (<14.0 g/kg DM) have been reported for silages of *G. vermiculophylla* [25], *F. vesiculosus* [26], *U. lactuca* [47] and *Ascophyllum nodosum* [44], but much greater lactate concentrations (up to 228 g/kg DM) have been shown for pre-wilted silages of *S. latissima* [25,26,44]. In well-preserved silages, lactic acid bacteria are active in degrading water-soluble carbohydrates and the lactate produced causes a decrease in pH that preserves the silage [41]. The low lactate contents observed in our study are consistent with the greater pH values compared with those reported in previous studies with *S. latissima* silages previously discussed [25,26,44]. Altogether, these results indicate that the activity of lactic acid bacteria in our silages was scarce, and that the good ensilability characteristics of *S. latissima* reported by other authors were not observed in our study. 

In unwilted *S. latissima* silages, the average L:DL-lactate ratio was 0.15 indicating that bacteria mainly producing D-lactate were responsible for most of the lactate production in these silages. By comparison, average L:DL-lactate ratio was 0.57 in unwilted *P. umbilicalis* silages. In silages made from terrestrial plants like grass, clover and maize whole crop, a L:DL-lactate ratio close to 0.50 (racemic) is found, and this was also expected for seaweed silages. Substances in the complex seaweed matrix might have inhibited the membrane oxidase used in L-lactate analysis causing the low L-lactate concentrations in *S. latissimi*; however, the recovery test of added L-lactate to seaweed silage samples showed complete recovery.

Pre-wilting of *P. umbilicalis* silages decreased (*p* ≤ 0.004) the production of all VFA detected (Table 2), except for valerate and caproate. In general, washing the seaweeds and adding FAC decreased (*p* < 0.05) concentrations of acetate, propionate, butyrate, isobutyrate and isovalerate of the unwilted silages. Similar or even greater VFA concentrations have been reported for silages of *G. vermiculophylla* [25], *U. lactuta* [44] *Laminaria digitata* [44]. The high VFA content (especially of butyrate) of the unwilted silages, together with their great NH_3_-N concentrations, seems to indicate a clostridial fermentation with proteolytic clostridia degrading the proteins to NH_3_-N [43]. However, in the pre-wilted silages acetate was practically the only VFA noticed, as the other VFA were detected in negligible amounts and only for some silage types. The low butyrate and NH_3_-N concentrations (<0.21 and 0.80 g/kg DM, respectively) would not indicate a clostridial fermentation in these silages. 

In contrast to that observed for *P. umbilicalis,* acetate and butyrate were the only VFA detected in *S. latissima* silages, and butyrate was only detected in the unwilted CON silages (Table 3). The CON silage also showed the greatest (*p* < 0.05) acetate concentration for unwilted silages, whereas there were no differences among silage types when *S. latissima* was pre-wilted. These results, together with the low NH_3_-N concentrations (<0.64 g/kg DM), seem to preclude a clostridial fermentation in *S. latissima* silages. Our results agree well with previous studies showing acetate concentrations ranging from 2.2 to 3.9 g/kg DM and very low or null levels of other VFA in pre-wilted *S. latissima* silages [25,26].

Ethanol concentrations in *P. umbilicalis* silages were decreased (*p* < 0.001) by pre-wilting to low values (≤0.36 g/kg DM), but they were not affected (*p* = 0.250) by silage type. In addition, very low levels of propanol, 2-butanol, ethylacetate and propylacetate (≤1.04 g/kg DM) were observed in all *P. umbilicalis* silages, being undetectable in most of the pre-wilted silages. Ethanol concentrations in *S. latissima* ranged from 0.050 to 2.14 g/kg DM, were not affected by either pre-wilting or silage type, and were lower than those reported previously (up to 47 g/kg DM) for *S. latissima* silages [26,44]. Similar to that observed for *P. umbilicalis* silages, concentrations of other alcohols and esters were negligible (≤0.018 g/kg DM) in *S. latissima* silages. In agreement with our results, ethanol was the main alcohol in silages of *F. vesiculosus*, *L. digitata* and *S. latissima* silages, and concentrations of other alcohols were minor in previous studies [26,44]. Ethanol content in silages is usually attributed to the activity of epiphytic, salt-tolerant yeast populations [47].

The differences observed in the characteristics of *S. latissima* and *P. umbilicalis* silages are yet to be elucidated, but they indicate different ensilability possibly due to differences in chemical composition, content in lactic acid bacteria, and buffering capacity, among others [44,47,48].

### 3.3. Chemical Composition and In Vitro Gas Production Parameters of the Seaweed Silages

Pre-wilting promoted limited changes on chemical composition of silages (Table 4 and Table 5), as the only changes observed were an increase in the NDF content for *P. umbilicalis* (*p* < 0.001; 156 vs. 334 g/kg DM; values averaged across silage types) and in the N content for *S. latissima* (*p* = 0.036; 8.90 vs. 9.10 g/kg DM). The increase in NDF content of *P. umbilicalis* might be due to losses of small particles through the holes of the net used in the pre-wilting process, as these particles probably had lower NDF content. In contrast, there were marked differences among silages type, and pre-wilting × silage type interactions (*p* ≤ 0.043) were observed for all chemical fractions of *P. umbilicalis.* As expected, washing decreased (*p* < 0.05) ash content, and for pre-wilted *P. umbilicalis* resulted in greater (*p* < 0.05) N, ADIN and ADF content and lower (*p* < 0.05) NDF content; however, when *P. umbilicalis* was not pre-wilted washing only decreased (*p* < 0.05) N and NDF content of the silages. The inclusion of FAC did not affect chemical composition of *P. umbilicalis*, with the exception of a decrease (*p* < 0.05; 324 vs. 234 g NDF/kg DM) in the NDF content of pre-wilted silages. Previous studies [49,50] have shown that formic acid can cause a decrease in NDF concentrations of ensiled plants due to acid hydrolysis.

Differences among the *S. latissima* silages were less marked than those observed for *P. umbilicalis* silages, and pre-wilting × silage type interactions were only observed for N content (*p* = 0.014). Washing decreased (*p* < 0.05) both ash and N contents, although differences for unwilted *S. latissima* silages did not reach the significance level. The use of FAC did not change chemical composition of *S. latissima* silages. Previous studies showed that ensiling *S. latissima* decreased their ash content [26,44], whereas Cabrita et al. [25] reported an increase in ash content in the silage. In our study, the ash content of both seaweeds in the silage was similar than that observed in the fresh biomass (Table 1). The N content in the silages of both seaweeds was similar to that of the fresh biomass (34.5 and 36.0 g N/kg DM for silages and fresh biomass of *P. umbilicalis*, respectively; 9.00 and 8.71 g N/kg DM for *S. latissima*; values averaged across treatments). This indicates that no N losses occurred during silage fermentation and it is in accordance with the low NH_3_-N content observed in the silage extracts. In agreement with our results, Cabrita et al. [25] reported no CP losses during silage fermentation of pre-wilted *S. latissima* for 9 weeks.

The NDF content of *P. umbilicalis* silages was lower than that in the fresh seaweed, especially in the unwilted silages. Cabrita et al. [25] observed similar NDF decreases during silage fermentation of the red seaweed *G. vermiculophylla*. These results, together with the lack of decrease in ADF content, might suggest that hemicellulose-like components were degraded during the silage fermentation process. Cabrita et al. [25] observed increases in acetate and butyrate content in the silages showing reduced NDF levels, which was attributed to fibre degradation during silage fermentation. In accordance with these results, greater concentrations of both acids were observed in the silages that showed decreased NDF levels compared with fresh *P. umbilicalis* (all silages except the pre-wilted CON; Table 2). The negative correlations between NDF content and both acetate (r = 0.699; *p* = 0.002; n = 18) and butyrate (r = 0.479; *p* = 0.050; n = 18) concentrations in the extracts of *P. umbilicalis* silages support this hypothesis.

On the contrary, the NDF content in all *S. latissima* silages was similar to that in the fresh biomass, and no relationships between NDF content and concentrations of acetate (*p* = 0.911) and butyrate (*p* = 0.344) were detected. Similarly, Cabrita et al. [25] observed no changes in NDF content during the silage fermentation of *S. latissima*. On the contrary, Campbell et al. [26] reported that NDF and ADF contents were 4.5 and 2.4 times lower, respectively, in *S. latissima* silages than in the fresh seaweed. These authors attributed the NDF and ADF losses to the degradation of laminarin and/or mannitol during the silage fermentation process, as they are the main storage carbohydrates in brown seaweeds [51] and can be used as substrate by the lactic acid bacteria during silage fermentation [52]. It should be noticed that the NDF and ADF content of the *S. latissima* ensiled in the study of Campbell et al. [26] was unusually high (384 and 184 g/kg DM, respectively), and the samples used by Cabrita et al. [25] and in our study had lower NDF and ADF levels (<130 and 66 g/kg DM, respectively). Differences in the carbohydrate content and profile of *P. umbilicalis* and *S. latissima* might be responsible for the different changes in fiber content observed in our study for both seaweeds, and for the contrasting results among studies in which the same seaweed was ensiled. Finally, it is worth to mention that lignin contents in *S. latissima* silages were similar to those reported previously [25,26], despite no lignin was detected in the fresh biomass of this seaweed. The presence of lignin in the silages might be due to protein-fiber interactions occurring during silage fermentation, similar to those produced during heating that result in increased ADIN proportions [35,53]. The greater ADIN proportions in the *S.latissima* silages compared with the fresh seaweed are consistent with this hypothesis.

The gas production kinetics of silages was measured as an index for their ruminal degradation, as the amount of gas produced is closely related to the amount of organic matter degraded by ruminal microorganism [37]. As shown in Table 4, pre-wilting of *P. umbilicalis* increased (*p* ≤ 0.022) the fractional rate of gas production (*c*), AGPR and DMED, and pre-wilting × silage type interactions were detected for these parameters (*p* ≤ 0.093). The greater PGP, *c*, AGPR and DMED values of the WASFAC silages compared with CON and WAS would indicate that FAC treatment increased the ruminal degradation of silages. These results are in accordance with the lower NDF levels in WASFAC compared with CON silages, as NDF has usually lower ruminal degradation than other compounds such as protein or soluble carbohydrates [53]. The gas production parameters of the silages were similar to those of the fresh *P. umbilicalis* for CON and WAS silages, but WASFAC silages had greater PGP, c, AGPR, DMED values, which indicates that FAC treatment increased the ruminal fermentation of the silages DM. 

In agreement with the results observed for *P. umbilicalis,* pre-wilting of *S. latissima* increased (*p* ≤ 0.006) *c* and AGPR values, but pre-wilting × silage type interactions (*p* ≤ 0.092) were detected for all gas production parameters excepting *c* (Table 5). Pre-wilting also increased (*p* = 0.026) the time until gas production started (*lag*) and decreased DMED (*p* = 0.029; 462 vs. 452 g/kg; values averaged across silage types) and PDMD values (915 vs. 894 g/kg), which might be due to the loss of small particles of high-degradable material during pre-wilting. However, the differences were low and no relevant in the practice. Including FAC in the *S. latissima* silages increased (*p* < 0.05) AGPR and DMED compared with the FAC-untreated silages. Altogether the results indicated that FAC could increase the ruminal degradation of seaweeds silages.

Samples of two good-quality silages [54] of perennial rye-grass were included in the in vitro incubations to be used as a reference (Table 6). Chemical composition of rye-grass silages differed markedly from that of seaweeds silages, as they had greater NDF and ADF content, their N content was lower than in *P. umbilicalis* silages but greater than in *S. latissima* silages. Chemical composition of the perennial rye-grass silages was similar to that previously reported for high-quality rye-grass silages [55,56]. The lower DMED of the seaweed silages compared with that of the rye-grass silages (59.9 and 86.1% of the rye-grass silage values for *P. umbilicalis* and *S. latissima*, respectively) indicates lesser nutritional quality of the seaweed silages, especially of those from *P. umbilicalis*. The differences observed in the DMED of the two seaweeds silages are probably related to their chemical composition, as brown seaweeds are richer in non-structural carbohydrates than red and green seaweeds and are therefore more degradable in the rumen [12,21]. In addition, the presence of complex carbohydrates and biocompounds in seaweeds might limit their ruminal fermentation, as specific enzymes are needed for their degradation [12], and other factors, such as the structure of the cell wall, can also limit the access of microbial enzymes to the substrates [57]. Interestingly, the DMPD of the *S. latissima* silages was similar to that of the rye-grass silages (905 and 915 g/kg, respectively; averaged values), although that *of P. umbilicalis* silages was lower (804 g/kg). These results disagree with the fact that PGP of the rye-grass silages was 3.0 and 1.4 times greater than in the *P. umbilicalis* and *S. latissima* silages, respectively (averaged values, 273, 90.3 and 196 mL/g DM). All seaweed silages had high content of ash (>110 and 252 g/kg DM for *P. umbilicalis* and *S. latissima* silages, respectively), that can disappear instantly from the bags during the incubations in the rumen or in vitro, causing an overestimation of their rumen degradability [58]. In contrast, ash is not fermented in the rumen and therefore does not contribute to gas production as measured in the present study.

## 4. Conclusions

Pre-wilting before ensiling is recommended for both *P. umbilicalis* and *S. latissima* seaweeds, as reduced the formation of effluent and the production of NH_3_-N and volatile fatty acids, indicating better silage quality. Washing the seaweeds before ensiling is advisable to reduce the ash content of the silages, although the effect on ruminal degradability can vary with the seaweed. The use of formic acid as a silage additive contributed effectively to reduce the pH and increased the ruminal degradability of silages. The estimated DM ruminal degradability of the *P. umbilicalis* and *S. latissima* silages was, as average, 59.9 and 86.1% of that in a high-quality rye-grass silage, respectively, indicating a medium-low nutritional value of these seaweed silages for ruminants. More studies are needed to optimize the conditions for good silage fermentation both seaweeds.

## Figures and Tables

**Table 1:** Chemical composition and in vitro gas production parameters of samples of fresh *Porphyra umbilicalis* and *Saccharina latissima* pre-ensiling.

	*Porphyra umbilical*		*Saccharina latissima*	
Item	Unwashed	Washed	Unwashed	Washed
Chemical composition ^1^				
DM (g/kg fresh matter)	215	198	137	144
Ash (g/kg DM)	210	152	290	259
N (g/kg DM)	34.8	37.2	9.19	8.23
ADIN (g/100 g total N)	1.50	1.16	3.68	3.52
NDF (g/kg DM)	443	428	94.2	102.0
ADF (g/kg DM)	27.6	25.8	65.7	61.3
Lignin	nd	nd	nd	nd
Gas production parameters ^2^				
PGP (mL/g DM)	80.3	85.3	185	195
*c* (%/h)	1.87	2.07	4.03	3.97
*Lag* (h)	0.15	0.0	2.90	2.28
AGPR (mL/h)	1.06	1.24	4.58	4.93
DMED (g/kg)	308	327	468	479
DMPD (g/kg)	814	811	922	921

^1^ DM: dry matter; N: nitrogen; ADIN: Acid detergent insoluble N; NDF: neutral detergent fiber; ADF: acid detergent fiber; nd: not detected; ^2^ PGP: potential gas production; *c*: fractional rate of gas production, *lag*: time before starting gas production, AGPR: average gas production rate until half of PGP was reached; DMED: DM effective degradability calculated for a rumen particulate outflow of 3% per h; DMPD: DM potential degradability after 120 h of in vitro incubation.

**Table 2:** Characteristics of *Porphyra umbilicalis* silages ^1^.

	Unwilted			Pre-Wilted				*p*-Value		
Item	CON	WAS	WASFAC	CON	WAS	WASFAC	SEM	Pre-Wilting	Silage Type	Pre-Wilting × Silage Type
Dry matter (DM; g/kg fresh matter)	181	166	170	666 ^b^	457 ^a^	439 ^a^	19.2	<0.001	<0.001	<0.001
**Effluent measures ^2^**										
Free juice	20.0 ^a^	31.3 ^c^	25.1 ^b^	nd ^4^	nd	nd	1.47	<0.001	0.008	0.008
Particles and particle-bound juice	80.0 ^c^	68.7 ^a^	74.9 ^b^	100	100	100	1.47	<0.001	0.008	0.008
**Parameters in silage extracts ^3^**										
pH	4.67 ^b^	4.54 ^b^	3.98 ^a^	5.75 ^c^	4.92 ^b^	3.78 ^a^	0.08	<0.001	<0.001	<0.001
NH_3_-N	5.30 ^b^	2.46 ^a^	1.74 ^a^	0.16	0.79	0.43	0.419	<0.001	0.006	0.001
Glucose	0.14	0.57	0.75	0.44	0.67	0.63	1.191	0.550	0.110	0.561
DL-lactate	2.26 ^a^	23.0 ^b^	10.0 ^a^	0.51^a^	14.2 ^b^	2.22 ^a^	3.760	0.070	0.002	0.611
L-lactate	1.35 ^a^	12.23 ^b^	5.87 ^a^	0.57 ^a^	8.61 ^b^	2.22 ^a^	1.93	0.115	0.001	0.704
Acetate	34.4 ^c^	25.7 ^b^	18.8^a^	0.97 ^a^	7.50 ^b^	3.33 ^a,b^	1.83	<0.001	0.008	<0.001
Propionate	33.0 ^b^	9.8 ^a^	4.2 ^a^	nd	nd	nd	2.19	<0.001	<0.001	<0.001
Butyrate	13.5 ^b^	7.09 ^a,b^	4.67 ^a^	nd	0.19	0.20	2.29	<0.001	0.193	0.167
Isobutyrate	0.57 ^b^	0.18 ^a^	0.16 ^a^	nd	nd	nd	0.11	0.004	0.139	0.139
Isovalerate	0.55 ^b^	0.21 ^a,b^	0.15 ^a^	nd	0.03	nd	0.11	0.008	0.222	0.188
Valerate	0.45	0.34	0.28	nd	nd	nd	0.20	0.053	0.918	0.918
Caproate	0.07	0.12	nd	0.02	nd	nd	0.06	0.242	0.571	0.595
Ethanol	3.34	3.65	2.16	0.04	0.36	0.10	0.50	<0.001	0.250	0.397
Propanol	0.92 ^b^	1.04 ^b^	0.14 ^a^	nd	0.02	nd	0.21	0.002	0.106	0.114
2-butanol	0.01 ^a^	0.13 ^b^	0.05 ^a^	nd	0.02	nd	0.03	0.026	0.050	0.198
Ethylacetate	0.12 ^b^	0.08 ^b^	0.02 ^a^	nd	0.01	nd	0.01	<0.001	0.020	0.021
Propylacetate	0.03 ^b^	0.02 ^b^	nd ^a^	nd	nd	nd	0.01	0.002	0.079	0.086
**Free amino acids**										
Alanine	6.05 ^a^	7.79 ^b^	5.32 ^a^	4.04 ^a^	7.34 ^b^	5.28 ^a^	0.412	0.029	<0.001	0.077
Proline	0.28 ^a^	1.01 ^b^	0.42 ^a^	0.16 ^a^	0.65 ^b^	0.32 ^a,b^	0.121	0.079	<0.001	0.516
Isoleucine	0.62 ^a^	0.82 ^b^	0.57 ^a^	0.12 ^a^	0.44 ^b^	0.23 ^a^	0.049	<0.001	<0.001	0.239
Leucine	1.25 ^a^	1.77 ^b^	1.08 ^a^	0.17 ^a^	0.77 ^b^	0.44 ^a^	0.101	<0.001	<0.001	0.122
Valine	1.42 ^b^	2.07 ^c^	1.09 ^a^	0.22 ^a^	1.02 ^b^	0.51 ^a^	0.102	<0.001	<0.001	0.019
Phenylalanine	0.40 ^a^	0.79 ^b^	0.47 ^a^	0.07 ^a^	0.36 ^b^	0.25 ^a,b^	0.089	<0.001	0.009	0.560

^a, b, c^ Within each variable and pre-wilting treatment, mean values in the same row with different superscripts differ (*p* < 0.05); ^1^ CON: seaweed unwashed and ensiled without any additive; WAS: seaweed washed and ensiled without any additive; WASFAC: seaweed washed and ensiled with 4 g of formic acid per kg seaweed; ^2^ % unless stated otherwise; ^3^ excepting pH values, g/kg dry matter (DM) unless stated otherwise; ^4^ nd: not detected.

**Table 3:** Characteristics of *Saccharina latissima* silages ^1^.

	Unwilted			Pre-Wilted				*p*-Value		
Item	CON	WAS	WASFAC	CON	WAS	WASFAC	SEM	Pre-Wilting	Silage Type	Pre-Wilting × Silage Type
Dry matter (DM; g/kg fresh matter)	161	136	134	405 ^a^	459 ^b^	390 ^a^	12.2	<0.001	0.040	0.020
**Effluent measures ^2^**										
Free juice	8.1 ^b^	3.7 ^a^	20.7 ^c^	nd ^4^	nd	nd	0.69	<0.001	<0.001	<0.001
Particles and particle bound juice	91.9 ^b^	96.3 ^c^	79.3 ^a^	100	100	100	0.69	<0.001	<0.001	<0.001
**Parameters in silage extracts ^3^**										
pH	4.87 ^b^	5.11 ^b^	3.73 ^a^	6.23 ^c^	5.84 ^b^	3.52 ^a^	0.12	<0.001	<0.001	<0.001
NH_3_-N	0.63 ^b^	0.29 ^a^	0.28 ^a^	0.25 ^c^	0.07 ^a^	0.16 ^b^	0.016	<0.001	<0.001	<0.001
Glucose	78.8 ^c^	47.5 ^b^	14.9 ^a^	40.4 ^b^	46.3 ^b^	6.22 ^a^	5.051	0.002	<0.001	0.008
DL-lactate	8.48 ^b^	7.98 ^b^	0.23 ^a^	0.22	0.13	nd	1.109	<0.001	0.004	0.005
L-lactate	0.25 ^a,b^	1.34 ^b^	0.06 ^a^	0.47	0.33	0.42	0.401	0.673	0.327	0.215
Acetate ^4^	18.3 ^b^	5.20 ^a^	5.36 ^a^	5.35	3.13	1.89	3.241	0.038	0.046	0.229
Butyrate	2.06 ^b^	nd ^a^	nd ^a^	nd	nd	nd	0.060	<0.001	<0.001	<0.001
Ethanol	1.99	1.27	0.85	0.07	2.14	0.05	0.582	0.220	0.142	0.094
Propanol	nd	nd	nd	0.004	nd	0.002	0.0018	0.195	0.586	0.586
2-Butanol	0.003 ^a^	0.008 ^b^	0.001 ^a^	0.001	nd	nd	0.0012	0.002	0.003	0.019
Ethylacetate	0.018	0.001 ^a^	0.002 ^a^	0.001	0.001	0.002	0.0035	0.060	0.059	0.042
**Free amino acids**										
Alanine	2.39 ^c^	1.69 ^b^	1.34 ^a^	2.35 ^b^	0.69 ^a^	0.64 ^a^	0.049	<0.001	<0.001	<0.001
Proline	0.23 ^c^	nd ^a^	0.07 ^b^	0.27 ^b^	0.11 ^a^	0.07 ^a^	0.021	0.019	<0.001	0.079
Isoleucine	0.25 ^b^	nd ^a^	nd ^a^	0.15 ^b^	nd ^a^	nd ^a^	0.012	<0.001	<0.001	<0.001
Leucine	0.29 ^b^	nd ^a^	nd ^a^	0.17 ^b^	nd ^a^	nd ^a^	0.011	<0.001	<0.001	<0.001
Valine	0.31 ^c^	0.13 ^b^	nd ^a^	0.22 ^b^	0.02 ^a^	nd ^a^	0.030	0.017	<0.001	0.185
Phenylalanine	0.19 ^c^	0.11 ^b^	nd ^a^	0.12 ^b^	nd ^a^	0.02 ^a^	0.022	0.019	<0.001	0.053

^a, b, c^ Within each variable and pre-wilting treatment, mean values in the same row with different superscripts differ (*p* < 0.05); ^1^ CON: seaweed unwashed and ensiled without any additive; WAS: seaweed washed and ensiled without any additive; WASFAC: seaweed washed and ensiled with 4 g of formic acid per kg seaweed; ^2^ % unless stated otherwise; ^3^ excepting pH values, g/kg dry matter (DM) unless stated otherwise; ^4^ No propionate, isobutyrate, isovalerate, valerate, caproate and propylacetate were detected in any silage; ^4^ nd: not detected.

**Table 4:** Chemical composition and gas production parameters of *Porphyra umbilicalis* silages ^1^.

	Unwilted			Pre-Wilted				*p*-Value		
Item	CON	WAS	WASFAC	CON	WAS	WASFAC	SEM	Pre-Wilting	Silage Type	Pre-Wilting × Silage Type
**Chemical composition** ^2^										
Ash (g/kg DM)	253 ^b^	119 ^a^	111 ^a^	224 ^b^	131 ^a^	138 ^a^	10.0	0.698	<0.001	0.043
N (g/kg DM)	35.0 ^b^	33.6 ^a^	34.1 ^a^	31.8 ^a^	36.0 ^b^	36.4 ^b^	0.462	0.224	0.004	<0.001
Acid detergent insoluble N (g/100 g total N)	1.33	1.69	1.96	0.25 ^a^	2.92 ^b^	3.04 ^b^	0.237	0.101	<0.001	0.002
Neutral detergent fiber (g/kg DM)	184 ^b^	129 ^a^	156 ^a,b^	455 ^c^	324 ^b^	234 ^a^	18.2	<0.001	<0.001	<0.001
Acid detergent fiber (g/kg DM)	37.4 ^a^	41.6 ^a^	51.9 ^b^	20.5 ^a^	55.2 ^b^	58.9 ^b^	4.5	0.954	<0.001	0.013
**Gas production parameters ^3^**										
PGP (mL/g DM)	89.1 ^a^	87.8 ^a^	95.7 ^b^	88.8^a^	85.7 ^a^	98.4 ^b^	1.59	0.266	<0.001	0.610
*c* (%/h)	1.59 ^a^	2.07 ^b^	2.14 ^b^	1.80 ^a^	2.05 ^b^	2.41 ^c^	0.072	0.006	<0.001	0.093
*Lag* (h)	0.01	0.05	0	0	0	0	0.022	0.274	0.409	0.409
AGPR (mL/h)	1.00 ^a^	1.24 ^b^	1.46 ^c^	1.15 ^a^	1.26 ^b^	1.70 ^c^	0.043	<0.001	<0.001	0.018
DMED (g/kg)	279 ^a^	331 ^b^	325 ^b^	298 ^a^	319 ^b^	356 ^c^	6.7	0.022	<0.001	0.008
Potential DM degradability (g/kg)	817	816	789	808	789	805	9.3	0.380	0.288	0.110

^a, b, c^ Within each variable and pre-wilting treatment, mean values in the same row with different superscripts differ (*p* < 0.05). ^1^ CON: seaweed unwashed and ensiled without any additive; WAS: seaweed washed and ensiled without any additive; WASFAC: seaweed washed and ensiled with 4 g of formic acid per kg seaweed; ^2^ no lignin was detected in any silage; ^3^ PGP: potential gas production; *c*: fractional rate of gas production, *lag*: time before starting gas production, AGPR: average gas production rate until half of PGP was reached; DMED: DM effective degradability calculated for a rumen particulate outflow of 3% per h; DMPD: DM potential degradability after 120 h of in vitro incubation.

**Table 5:** Chemical composition and gas production parameters of *Saccharina latissima* silages ^1^.

	Unwilted			Pre-Wilted				*p*-Value		
Item	CON	WAS	WASFAC	CON	WAS	WASFAC	SEM	Pre-Wilting	Silage Type	Pre-Wilting × Silage Type
**Chemical composition**										
Ash (g/kg DM)	278	264	268	302 ^b^	252 ^a^	264 ^a^	9.6	0.735	0.017	0.200
N (g/kg DM)	9.58	8.88	8.25	10.2 ^b^	8.34 ^a^	8.75 ^a^	0.264	0.036	0.009	0.014
Acid detergent insoluble N (g/100 g total N)	12.9	12.5	12.9	12.6	13.1	13.0	1.37	0.477	0.726	0.549
Neutral detergent fiber (g/kg DM)	105	110	118	127	110	111	6.0	0.304	0.570	0.073
Acid detergent fiber (g/kg DM)	81.4	86.5	92.5	84.7	83.0	85.6	4.1	0.492	0.353	0.474
Lignin (g/kg DM)	10.6	8.77	7.73	16.3	10.2	9.30	2.81	0.129	0.216	0.826
**Gas production parameters ^2^**										
PGP (mL/g DM)	191	195	196	185 ^a^	206 ^b^	203 ^b^	2.4	0.054	<0.001	0.002
*c* (%/h)	3.36 ^a^	3.71 ^b^	3.59 ^ab^	3.73 ^a^	4.03 ^b^	3.64 ^a^	0.102	0.003	0.003	0.192
*Lag* (h)	1.32 ^a^	2.90 ^b^	1.43 ^a^	2.66 ^b^	3.66 ^c^	1.01 ^a^	0.301	0.026	<0.001	0.015
AGPR (mL/h)	4.36 ^a^	4.47 ^ab^	4.72 ^b^	4.34 ^a^	4.89 ^b^	5.04 ^b^	0.103	0.006	<0.001	0.088
DMED (g/kg)	453 ^a^	450 ^a^	483 ^b^	439 ^a^	430 ^a^	486 ^b^	5.5	0.029	<0.001	0.092
Potential DM degradability (g/kg)	898 ^a^	914 ^b^	934 ^c^	878 ^a^	880 ^a^	925 ^b^	4.3	<0.001	<0.001	0.041

^a, b, c^ Within each variable and pre-wilting treatment, mean values in the same row with different superscripts differ (*p* < 0.05). ^1^ CON: seaweed unwashed and ensiled without any additive; WAS: seaweed washed and ensiled without any additive; WASFAC: seaweed washed and ensiled with 4 g of formic acid per kg seaweed; ^2^ PGP: potential gas production; *c*: fractional rate of gas production, *lag*: time before starting gas production, AGPR: average gas production rate until half of PGP was reached; DMED: DM effective degradability calculated for a rumen particulate outflow of 3% per h; DMPD: DM potential degradability after 120 h of in vitro incubation.

**Table 6:** Chemical composition of samples of early and late first cut of pre-wilted perennial ryegrass silages.

	Pre-Wilted Perennial Ryegrass Silage	
Item	Early First Cut	Late First Cut
**Chemical composition ^1^**		
DM (g/kg fresh matter)	350	350
Ash (g/kg DM)	77.1	62.9
N (g/kg DM)	18.0	14.5
ADIN (g/100 g total N)	0.0	1.10
NDF (g/kg DM)	389	438
ADF (g/kg DM)	186	227
Lignin (g/kg DM)	5.80	6.14
**Gas production parameters ^2^**		
PGP (mL/g DM)	271	274
*c* (per h)	5.70	4.81
*Lag* (h)	1.79	1.54
AGPR (mL/h)	9.67	8.56
DMED (g/kg)	548	513
DMPD (g/kg)	930	899

^1^ DM: dry matter; OM: organic matter; N: nitrogen; ADIN: acid detergent insoluble N; NDF: neutral detergent fiber; ADF: acid detergent fiber; Lignin: cellulose solubilization by sulfuric acid after extraction with acid detergent; ^2^ PGP: potential gas production; *c*: fractional rate of gas production, *lag*: time before starting gas production, AGPR: average gas production rate until half of PGP was reached; DMED: DM effective degradability calculated for a rumen particulate outflow of 3% per h; DMPD: DM potential degradability after 120 h of in vitro incubation.
